# Subtalar Dislocations

**DOI:** 10.5435/JAAOSGlobal-D-21-00295

**Published:** 2021-12-22

**Authors:** Siddhartha Sharma, Sandeep Patel, Mandeep S. Dhillon

**Affiliations:** From the Department of Orthopedics, Postgraduate Institute of Medical Education and Research, Chandigarh, India.

## Abstract

Subtalar dislocations are uncommon injuries that involve disruption of the talocalcaneal and talonavicular joints. Whereas medial subtalar dislocations are usually caused by low-energy mechanisms and are reducible by closed means, lateral subtalar dislocations occur due to high-energy trauma, have associated foot injuries, and may require open reduction. Good outcomes can be expected for low-energy medial dislocations, whereas high-energy dislocations have guarded outcomes. Hindfoot deformity and chronic instability can result from nonanatomic reduction and inadequate stabilization. Arthrosis of the subtalar joint can occur despite anatomic reduction and is attributable to the cartilage damage at the time of injury.

Subtalar dislocation, also termed variably as peritalar, subastralgar, or talocalcaneonavicular dislocation, is characterized by concurrent dislocations of the subtalar (talocalcaneal) and the talonavicular joints. These injuries can be broadly grouped into two distinct patterns, although other, lesser common variants have also been reported. Medial subtalar dislocations are more common, occur due to low-energy trauma, and generally have good functional outcomes. In stark contrast, lateral subtalar dislocations are less common, occur due to high-energy trauma, and have guarded outcomes.^1^ Anatomic reduction and stabilization of the subtalar joint (STJ) and optimal management of any associated foot injuries are the key to good outcomes but do not necessarily prevent arthrosis.^2^

## Anatomy

The literature on the anatomy of the STJ complex is variable. Whereas a section of the literature considers the posterior articulation between the talus and calcaneus as the STJ, other reports include the anterior talocalcaneonavicular and even the calcaneocuboid joints as a part of the STJ complex.^3^ From an anatomic perspective, three distinct components of the STJ complex can be identified.^3,4^ The posterior part of the STJ complex consists of the saddle-shaped articulation between the posterior facets of the talus and calcaneus; this is termed the talocalcaneal joint (TCJ). The anterior part, termed the talocalcaneonavicular joint (TCNJ), is formed by the articulations between the anterior and middle facets of the talus and calcaneus, the talar head, and the medial aspect of the calcaneonavicular ligament. The TCNJ is also called the coxa or the acetabulum pedis because of its anatomic and developmental similarities with the hip joint. The convex talar head resembles the femoral head and concave articular surfaces of navicular and anterior and middle facets of the calcaneus, the acetabulum. The interosseous canal between the talus and calcaneus forms the middle part of the STJ complex and separates the anterior and posterior parts. This canal is conical and consists of the broad sinus tarsi anterolaterally and the narrow canalis tarsi posteromedially. The interosseous canal houses the interosseous ligament complex, which is a key stabilizer of the STJ complex.^3^

Stability of the STJ is provided by its bony architecture and dense ligament complex. The ball and socket nature of the TCNJ imparts inherent osseous stability. Stabilizers of the STJ complex can be grouped into medial, lateral, anterior, and interosseous ligaments for simplicity.

**Figure 1:**
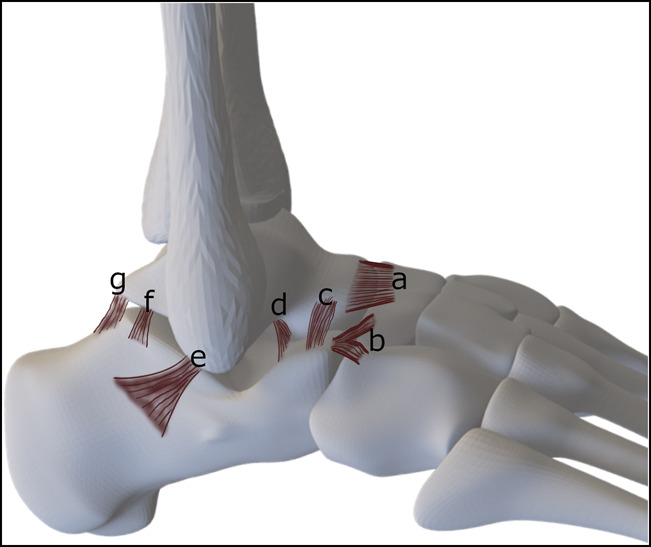
Ligaments of the subtalar joint, lateral aspect. (**a**) Lateral talonavicular ligament, (**b**) bifurcate ligament, (**c**) cervical ligament, (**d**) anterior talocalcaneal ligament, (**e**) calcaneofibular ligament, (**f**) lateral talocalcaneal ligament, and (**g**) posterior talocalcaneal ligament.

**Figure 2:**
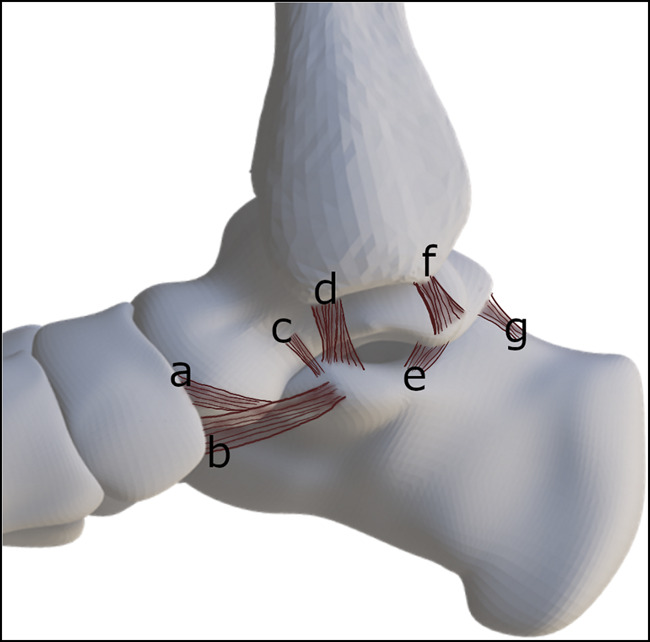
Ligaments of the subtalar joint, medial aspect. The superficial deltoid ligament is not depicted. (**a**) Talonavicular ligament, (**b**) superomedial band of the spring ligament, (**c**) medial talocalcaneal ligament–anterior arm, (**d**) tibiocalcaneal band of the deltoid ligament, (**e**) medial talocalcaneal ligament–posterior arm, (**f**) tibiotalar band of the deltoid ligament, and (**g**) posterior talocalcaneal ligament.

On the lateral aspect, the calcaneofibular ligament is the most important stabilizer of the STJ in inversion. The anterior, lateral, and posterior talocalcaneal ligaments are small, inconsistent, and offer little support by themselves (Figure 1). On the medial side, the tibiocalcaneal part of the deltoid ligament complex provides stability in eversion (Figure 2). Furthermore, the spring ligament and the talonavicular ligaments support the medial aspect of the talonavicular joint. The spring ligament comprises the broad superomedial ligament and the smaller, inferior plantar and medial plantar oblique ligaments. At the anterior aspect, the bifurcate ligament stabilizes both the talocalcaneonavicular and the calcaneocuboid joints. Finally, the interosseous ligament complex, consisting of five different ligaments (lateral and medial roots of the inferior extensor retinaculum, cervical ligament, and the interosseous talocalcaneal ligament), stabilizes the STJ in both inversion and eversion.^3^ In addition to the ligaments, stability is augmented by capsules of the talocalcaneal and the TCNJ.

The talus has a complex blood supply and receives contributions from the posterior tibial, anterior tibial, and the peroneal arteries. The deltoid branch of the posterior tibial artery supplies most of the talar body and medial part of the talar neck. The perforating branches of the dorsalis pedis artery enter the talar neck through its dorsal surface and supply the talar neck and the talar head. The peroneal artery has the least contribution, and supplies, the lateral part of the talar neck.^5^

## Biomechanics

Although the TCJ and the TCNJ are anatomically distinct joints with separate capsules, they function as a single unit.^4^ From a functional and biomechanical perspective, the STJ is considered a uniaxial joint with triplanar motion. As described by Manter et al,^6^ the STJ axis is directed medially, anteriorly, and superiorly. Movements of the STJ can be described in the frontal, sagittal, and transverse (coronal) planes. Frontal plane movements are varus and valgus and describe the movement of the calcaneus relative to the talus in the medial and lateral direction, respectively. Sagittal plane movements are dorsiflexion and plantar flexion and represent the upward and downward movement of the anterior end of the calcaneus, respectively. Transverse plane movements are adduction and abduction and describe the movement of the forefoot and midfoot in the medial and lateral directions relative to the hindfoot. Supination and pronation are essentially movements of the forefoot. Supination describes the position of the foot where the medial border is lifted off the ground, and pronation occurs when the lateral border of the foot is off the ground. Mosca^7^ compares the STJ motion to the act of wringing a towel and describes the subtalar motion in terms of movement of the acetabulum pedis around the head of the talus. Inversion is a combination of plantar flexion and varus of the heel along with adduction and supination of the forefoot. It may be thought of simply as the down and in position. In contrast, eversion is a combination of valgus and dorsiflexion of the heel, along with abduction and pronation of the forefoot; this may be thought of as the up and out position.^4,7^

The complex movements of the STJ contribute to the midtarsal joint locking mechanism, which is an essential component of the gait cycle and helps maintain the foot's flexibility. During the stance phase, the axes of the subtalar and midfoot joints become parallel, increasing the sagittal motion of the forefoot.^4^ This unlocks the foot and allows it to adapt to uneven surfaces in the stance phase. On the contrary, the axes of the subtalar and midfoot joints converge during propulsion. This motion locks the forefoot and makes it a rigid lever, allowing the triceps surae to generate adequate power for push-off.^4,7^

Joint kinetics of the STJ has been studied in detail. Wagner et al^8^ showed that the contact pressures in the TCJ (posterior facet) were highest in inversion. However, contact pressures in the TCNJ were not found to be affected by the foot position. Reeck et al^9^ demonstrated that the contact area and the contact pressure in the TCJ were higher in the toe-off position compared with the foot-flat position.

In summary, the STJ inverts during the toe-off phase, decreasing foot motion while increasing the TCJ contact pressures. It everts during the midstance phase in sharp contrast, allowing for greater flexibility and lowering TCJ contact pressures.

## Epidemiology

Judey and Dufaurest were the first to describe subtalar dislocation in 1811.^10^ These are rare injuries and are thought to account for <1% of all dislocations, although accurate epidemiologic data lack.^11,12^ In a literature review,^11^ the etiology was traffic accidents in 43.7% of all cases, falls in 32.9%, sports injuries in 13.9%, sprains in 5.3%, and other causes in 4.2%. Males were more commonly affected than females, and the dislocations occurred more frequently on the right side.^11^

## Classification

In 1853, Broca^13^ classified subtalar dislocations as medial, lateral, and posterior, depending on the position of the dislocated foot with respect to the talus. An anterior type was added later by Malgaigne and Henke.^14^ Medial subtalar dislocations account for approximately 75% of all cases and are characterized by medial dislocation of the foot and the heel in relation to the talus. Lateral dislocations account for 17% to 26% of all cases; the heel and foot dislocate laterally with respect to the talus.^15,16^ Anterior and posterior dislocations are rare injuries characterized by anterior and posterior dislocation of the foot and heel in relation to the talus, respectively.^17,18,19,20^

## Pathomechanics

Medial subtalar dislocations result from forced inversion of the plantarflexed foot; these generally result from low-energy trauma. In this position, the talar neck hinges and rotates around the sustentaculum tali, resulting in rupture of the lateral talonavicular joint capsule and ligaments, followed by the subtalar ligaments. The inversion force may also result in fracture of the posterior process of the talus, which is a common occurrence with medial subtalar dislocations. The talar neck may break off the sustentaculum tali in rare cases as it swivels around this pivot.^12,16^

In contrast, lateral subtalar dislocations are produced by high-energy trauma and are the result of forced eversion of a dorsiflexed foot. In this position, the talar head pivots around the anterior process of calcaneus, resulting in rupture of the talonavicular and subtalar ligaments and joint capsules. The same force may also result in partial or complete rupture of the deltoid ligament. An impaction fracture of the talar head or a push-off fracture of the anterior process of the calcaneus may occur as the soft talar head rotates around the anterior process of the calcaneus. Because of the high-energy mechanism of trauma, lateral subtalar dislocations often present with associated foot injuries, such as fractures of the ankle, talus, cuboid, cuneiforms, navicular, and metatarsals.^12,16,21^ Posterior subtalar dislocations are caused by forced plantar flexion of the foot, whereas anterior subtalar dislocations result from anterior traction to the foot, with the leg in a fixed position.^17,18,19,20^

Leitner et al^17^ think that a subtalar dislocation represents the first stage of a total talar dislocation, wherein the talus dislocates from all its articulations. In a medial total talar dislocation, forced plantar flexion causes the foot to dislocate from the ankle joint. Next, adduction and internal rotation forces cause the foot to rotate 90° along its vertical and longitudinal axis, dislocating the talus from the talocalcaneal and talonavicular joints. Finally, the displacing foot returns to its position leaving the talus dislocated laterally. Similarly, eversion forces may lead to a lateral total talar dislocation. Both of these are high-energy mechanisms.^22^

## Diagnosis

**Figure 3:**
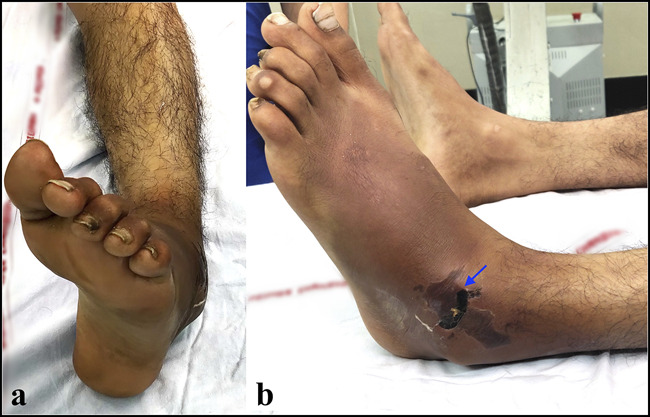
Clinical presentation of a 34-year-old man with medial subtalar dislocation who presented 12 hours after injury. **A,** Note the acquired clubfoot deformity. **B,** The talar head has tented the skin over the anterolateral aspect of the ankle with impending skin necrosis (arrow).

**Figure 4:**
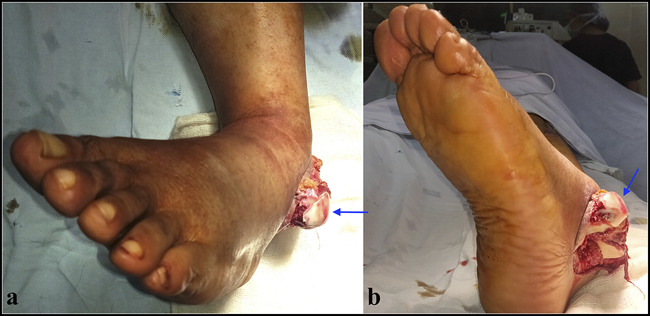
Open irreducible medial subtalar dislocation in a 54-year-old man after a road accident. **A,** Top view. (**B**) Side view. The talar head (arrow) is visible through the large lateral wound.

Medial subtalar dislocations result in an obvious foot deformity, likened to an acquired clubfoot. The foot is dislocated beneath the talus, the heel is displaced medially, and the forefoot is adducted and plantarflexed. The talar head is often palpable laterally (Figure 3). In contrast, lateral subtalar dislocation results in an acquired flatfoot deformity. The heel is displaced laterally, and the forefoot is abducted (Figure 4). Ecchymosis and swelling may be noted around the midfoot and hindfoot. Deep pain and limitation of passive range of motion of the subtalar and talonavicular joints are also noted. Note must be made of any wounds over the foot, and the distal neurovascular structures must be carefully examined. Anterior and posterior subtalar dislocations may not present with an obvious foot deformity. Hence, it is essential to evaluate these injuries carefully by radiography.^16,21^

**Figure 5:**
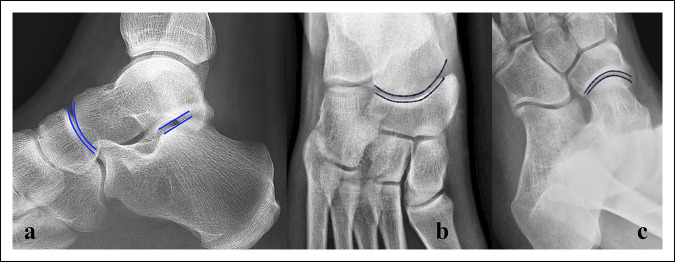
Normal radiology of the subtalar and talonavicular joints. (**A**) Lateral view: note the congruency of the talonavicular joint (head in cup appearance). The articular surfaces of the posterior facet of the calcaneus and talus are also noted to be parallel. **B,** AP view and (**C**) oblique view of the foot. Note the congruence of the talonavicular joint

**Figure 6:**
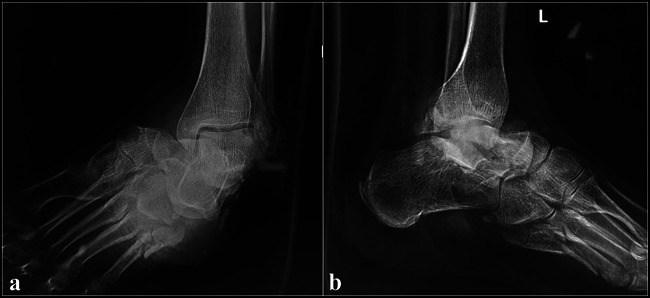
Radiographs of the medial subtalar dislocation case (presented in Figure 3). **A,** AP view. (**B**) Lateral view. Medial dislocation of the foot under the talus and absence of congruence of the talonavicular and subtalar joints are noted. The comminuted fifth metatarsal fracture is also noted.

**Figure 7:**
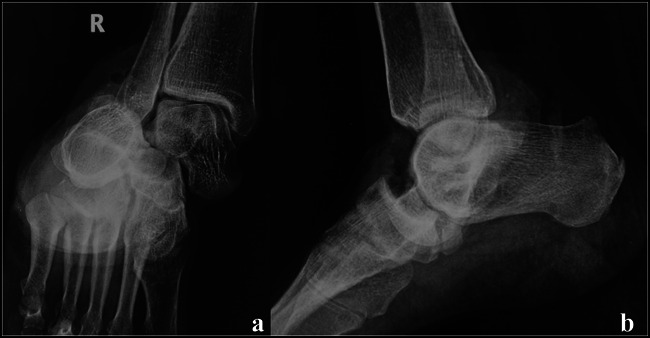
Radiographs of a 45-year-old woman with lateral subtalar dislocation case. **A,** AP view. (**B**) Lateral view. Lateral dislocation of the foot under the talus and absence of congruence of the talonavicular and subtalar joints are noted.

The diagnosis of a subtalar dislocation can be made on plain radiographs in most cases. The AP projection of the ankle will show medial or lateral dislocation of the foot beneath the talus. The lateral projection of the ankle will reveal loss of the normal talonavicular articulation. In a normal foot, the convexity of the talar head matches the concavity of the proximal articular surface of the navicular, the so-called head within the cup appearance (Figure 5). This relationship is disrupted in a subtalar dislocation. Disruption of the STJ may also be evident on the lateral view, which is visualized as a lack of parallelism between the articular surfaces of the talus and calcaneus. On an AP projection of the foot, disruption of the talonavicular joint may be noted as disruption of the head in cup relationship, akin to what is seen in the lateral view (Figures 6 and 7).^16,23^

**Figure 8:**
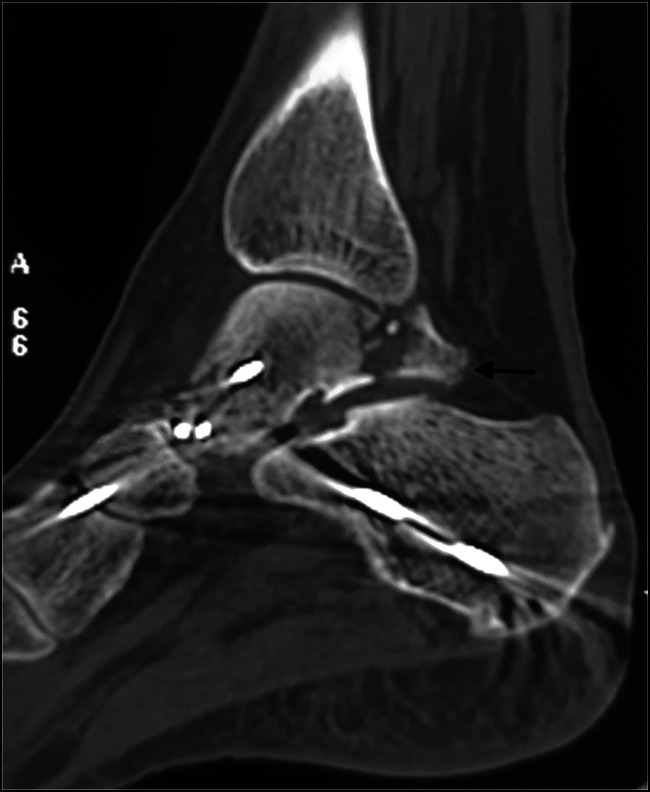
CT scan obtained after closed reduction and percutaneous pinning of a medial subtalar dislocation, which was unstable after reduction. Note the posterior process fracture extending into the subtalar joint. Large fragments such as these need to be fixed.

**Figure 9:**
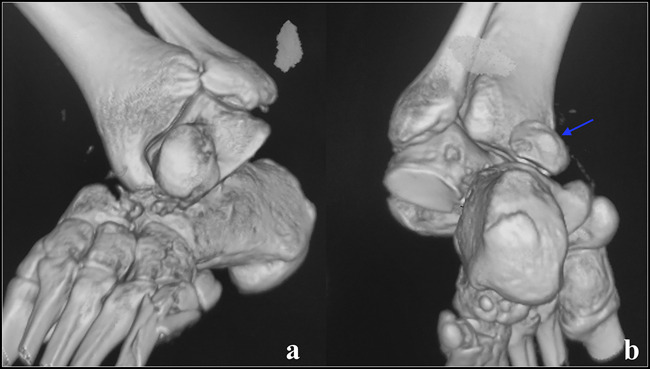
CT scan with volumetric three-dimensional reconstruction of a patient with irreducible medial subtalar dislocation. There is loss of articulation of the talonavicular and subtalar joints. The posterior process fracture is also noted (arrow).

A CT scan should be obtained for all subtalar dislocations because associated injuries may be present in up to 60% of the cases.^23^ If closed reduction can be performed, this is done first. A postreduction CT helps assess the congruency of the subtalar and talonavicular joints and any associated injuries that might require surgical intervention (Figures 8 and 9). In irreducible injuries, the CT scan may reveal osseous obstacles to reduction, such as an interposed osteochondral fragment or impaction of the navicular against the talar head.^16^

## Treatment

Subtalar dislocations should be reduced on an emergent basis. This reduces the risk of damage to soft tissues and neurovascular structures and the cartilage of the subtalar and the talonavicular joints. Closed reduction can usually be achieved in medial peritalar dislocations, whereas lateral subtalar dislocations are generally irreducible by closed means.^12,16,24^

## Closed Reduction

Adequate muscle relaxation is critical to achieving closed reduction. If the patient presents immediately after the injury, the reduction can be attempted under sedation. However, patients often present after a few hours, and general or regional anesthesia is necessary to achieve good muscle relaxation.^16^

**Figure 10:**
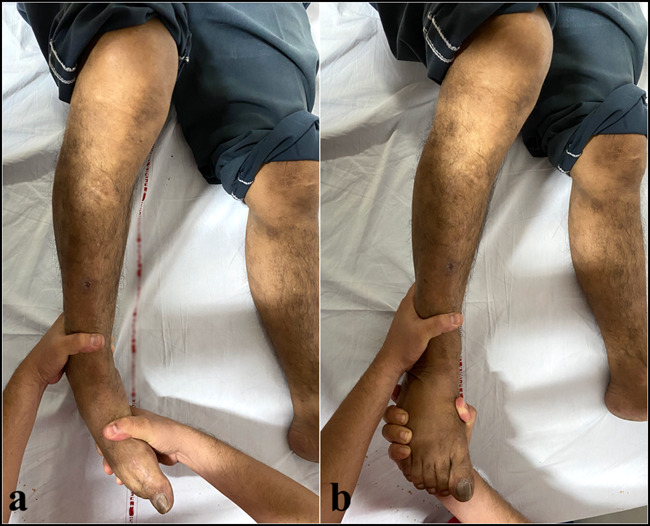
Closed reduction of a medial subtalar dislocation. **A,** The knee is flexed, and the dislocation is unlocked by increasing the deformity. **B,** Traction is applied to the heel, and the foot is brought into eversion gently. Direct pressure on the talar head is applied (not shown in the picture).

The closed reduction maneuver for a medial subtalar dislocation proceeds as follows. The gastrocnemius-soleus complex must be relaxed to decrease its pull on the calcaneus; this is achieved by flexing the knee to 90°. The calcaneus is generally locked under the talus, and it is necessary to unlock it by exaggerating the deformity. This is done by plantar flexion and inversion of the heel. Next, longitudinal traction is applied to the heel, and countertraction is applied to the leg. The foot is brought into dorsiflexion and eversion, and gentle pressure is applied over the palpable head of the talus. This results in reduction, accompanied by an audible or palpable click (Figure 10).^16,21^

For lateral subtalar dislocations, the gastrocnemius-soleus complex is relaxed by flexing the knee to 90°. The calcaneus is unlocked by dorsiflexion and eversion of the heel. Longitudinal traction on the heel, followed by gentle inversion, plantar flexion, and direct pressure on the talar head, accomplishes the reduction. However, these dislocations may be irreducible in up to 60% of the cases, and open reduction is needed.^16,21^

For the rare anterior and posterior subtalar dislocations, the gastrosoleus is relaxed by flexing the knee. Axial traction is applied to the heel. Anterior and posterior translation of the foot is then performed to reduce posterior and anterior dislocations, respectively.

Immobilization after closed reduction can be achieved in a below-knee splint or brace. If marked instability is noted, Kirschner wire stabilization of the STJ may be necessary. Postoperative radiographs are performed to assess the congruency of reduction. As discussed earlier, a CT scan is done in all cases after closed reduction to verify the accuracy of reduction and detect any associated fractures that might warrant surgical intervention.

For dislocations that are stable after reduction, full weight is permitted in a cast or a walker boot, which is maintained for 6 weeks. Range of motion exercises may be initiated after 3 to 4 weeks for isolated, low-energy dislocations that are deemed stable after closed reduction. Any Kirschner wires that have been used for stabilization are also removed after 3 to 4 weeks. Longer period of immobilization (6 weeks or more) and partial weight bearing may be needed for unstable high-energy dislocations that have associated foot injuries. Physical therapy focuses on regaining the complete range of motion at the ankle and STJs.^16^

## Irreducible Dislocations and Open Reduction

Closed reduction may be unsuccessful in up to 14% of all cases; several anatomic structures could contribute to the irreducibility.^22^ Irreducibility by closed means is more common in lateral subtalar dislocations. In medial subtalar dislocations, irreducibility is generally the result of buttonholing of the talar head through the capsule of the talonavicular joint or entrapment within the extensor retinaculum, extensor tendons, or the extensor digitorum brevis muscle. Rarely, interposition of the deep peroneal nerve and dorsalis pedis artery has also been demonstrated as potential blocks to the closed reduction of medial subtalar dislocations.^16,25,26^ Another irreducible variety is the locked dislocation wherein the navicular is impacted into the talar neck.^16^

Irreducibility in lateral subtalar dislocations is usually attributable to entrapment of the tibialis posterior tendon or the flexor hallucis longus tendon around the talar head.^16,25^ Interposed osteochondral fragments can also result in irreducibility and should be sought if there are no soft-tissue obstacles to reduction.^16,25^

**Figure 11:**
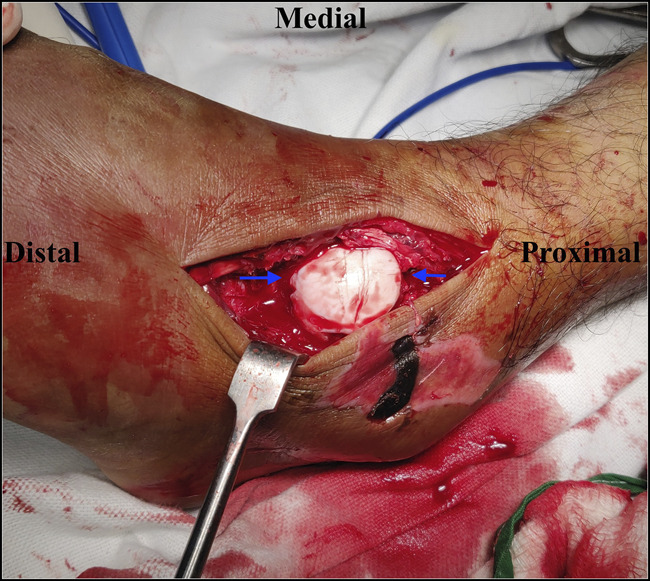
Open reduction of irreducible medial dislocation (presented in Figures 3 and 6). Note buttonholing of the talar head through the lateral talonavicular joint capsule. Once the capsule is divided, reduction can be achieved readily.

**Figure 12:**
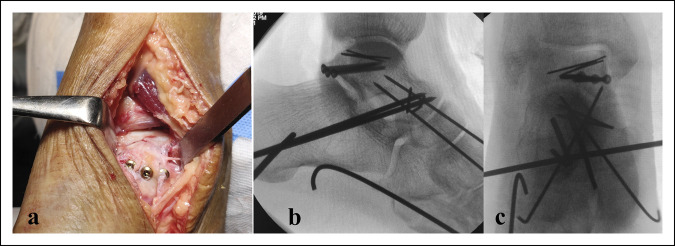
Fixation of an associated posterior process fracture after open reduction of a medial subtalar dislocation (presented in Figure 4). **A,** Posteromedial approach, showing fixation of the fracture fragment. **B** and **C,** Intraoperative lateral and AP views, showing the reduction and fixation.

When indicated, open reduction may be accomplished through an incision centered over the palpable dislocated talar head. After obstacles to reduction are identified and addressed, reduction is achieved easily (Figure 11). The subtalar and talonavicular joints are irrigated to ensure that they are free from small bony fragments and cartilage debris. If the reduction is unstable, it may be held in place by Kirschner wires across the STJ and talonavicular joint. Care must be taken not to place the Kirschner wires through the posterior facet of the STJ. Once the dislocation has been addressed, attention is turned to concomitant talar injuries. Fractures of the lateral process and posterior process of the talus may need to be fixed if these are large and involve a significant part of the STJ. This may often necessitate an additional surgical approach (Figure 12). Smaller fragments can be left alone or excised. Impaction of the talar head is addressed by elevation and bone grafting. If there are any associated foot injuries, these are also addressed on their merit.

## Open Subtalar Dislocations

Approximately 20% to 25% of all subtalar dislocations are open^11^; these are managed on the lines of other open injuries. Early institution of broad-spectrum antibiotics and meticulous wound débridement and lavage are the cornerstones of management. Temporary ankle spanning external fixation may be needed in the presence of large wounds to facilitate wound inspection and change of dressings.

## Complications and Outcomes

**Figure 13:**
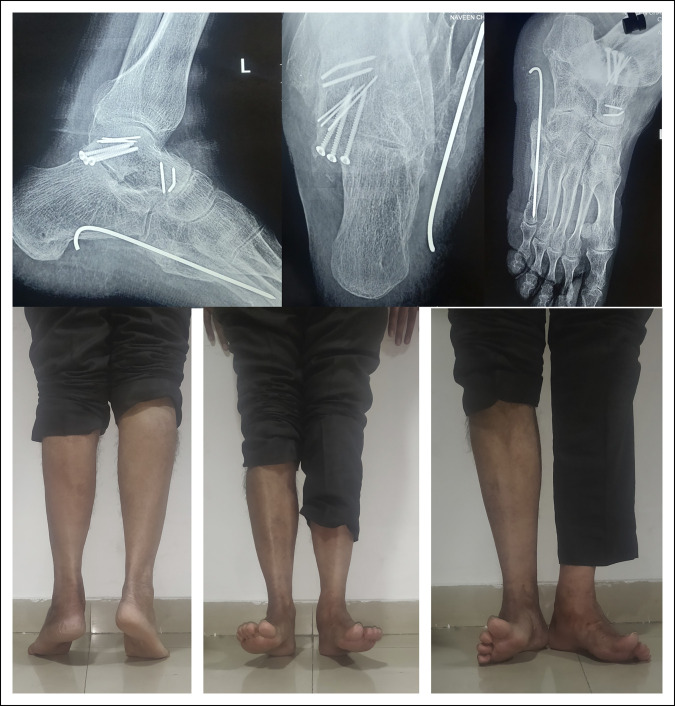
One-year follow-up of a patient with open irreducible medial subtalar dislocation (presented in Figures 4 and 12). The posterior process fracture has healed. There is no subtalar or talonavicular arthrosis. There is excellent range of motion at the ankle. Subtalar movements (inversion and eversion) are restricted.

It was traditionally thought that lateral subtalar dislocations have poor outcomes and medial dislocations have excellent outcomes^12,27^; however, this view has been challenged.^11,24,28^ The final outcome depends on several factors, including the severity of the trauma, articular cartilage damage, need for open reduction, prolonged immobilization, and associated foot injuries. Isolated, low-energy dislocations that are stable after closed reduction and are mobilized early can be expected to have an excellent prognosis^24,28,29^ (Figure 13). However, results can be expected to worsen as the severity of trauma increases. Osteonecrosis of the talus is seen in zero to 10% of closed dislocations and in up to 50% of open dislocations.^11,24^ Posttraumatic arthritis is perhaps the most common complication of subtalar dislocations and invariably involves the STJ, the reported rates varying from 40% to 89%.^11,24,30^ Midfoot arthritis is much less common. Patients with radiographic evidence of subtalar arthritis may not necessarily be symptomatic. In those who have troublesome subtalar arthritis, subtalar fusion may be needed. Chronic instability of the STJ is rare and attributable to inadequate immobilization. Mild to moderate instability may be managed with orthoses and muscle strengthening or ligamentoplasty. Gross instability with painful arthrosis of the STJ would necessitate subtalar arthrodesis.^16^

## Summary

Subtalar dislocations can occur due to low- or high-energy injury mechanisms. Up to 60% of these injuries have associated fractures. Hence, a CT scan is indicated to avoid the sequelae of missed injuries. Low-energy, isolated medial subtalar dislocations that can be reduced by closed means have the best prognosis. On the other hand, high-energy and open dislocations that have associated foot injuries fare worse. Subtalar arthrosis is the commonest complication; however, all patients with subtalar arthrosis do not require surgical intervention.
